# Clinical characteristics leading to misdiagnosis of abdominal tuberculosis in children: a systematic review and meta-analysis

**DOI:** 10.3389/fped.2025.1616608

**Published:** 2025-09-02

**Authors:** Mohd Jaish Siddiqui, Abhishek Karmacharya, Xuemeng Wan, Yu Zhu, Chaomin Wan, Shuanghong Luo

**Affiliations:** ^1^ West China Second University Hospital, Sichuan University, Chengdu, China; ^2^ Rapti Academy of Health Sciences, Ghorahi, Dang, Nepal

**Keywords:** abdominal tuberculosis, children, misdiagnosis, diagnostic delay, clinical characteristic, meta-analysis

## Abstract

**Introduction:**

Abdominal tuberculosis (ATB) in children is an uncommon form of extrapulmonary tuberculosis that often presents with non-specific symptoms. These features frequently overlap with other abdominal conditions, increasing the risk of misdiagnosis or delay in diagnosis. Although individual case series have reported such diagnostic challenges, a pooled analysis of clinical characteristics associated with misdiagnosis has not been previously conducted. This study aimed to identify the clinical characteristics that contribute to the misdiagnosis of ATB in children through a systematic review and meta-analysis.

**Methods:**

We conducted a systematic review and meta-analysis following PRISMA 2020 guidelines. A comprehensive literature search was carried out using PubMed, Web of Science, and Google Scholar for studies published between 1900 and 2024. Eligible studies were pediatric case series that included confirmed ATB cases and provided information on initial misdiagnosis or diagnostic delays. Patients who were misdiagnosed or delayed in diagnosis were categorized under the “misdiagnosed” group. Data were extracted on presenting clinical features, and odds ratios (ORs) with 95% confidence intervals (CIs) were calculated using Review Manager (RevMan 5.4). Heterogeneity was assessed using the *I*^2^ statistic, and publication bias was evaluated using funnel plots.

**Result:**

Seven studies met the inclusion criteria, comprising a total of 60 pediatric ATB cases. Among them, 24 were classified as misdiagnosed and 36 were diagnosed without delay. No clinical characteristics were statistically significantly associated with misdiagnosis. Although ascites and abdominal distension were more frequently observed in misdiagnosed cases, overall heterogeneity was low across most outcomes.

**Conclusions:**

Clinical characteristics alone are not reliable indicators for diagnosing ATB. Ascites and abdominal distension may increase the risk of misdiagnosis, underscoring the importance of early suspicion and timely diagnostic evaluation in TB-endemic regions.

## Introduction

Tuberculosis (TB) remains a major global health threat, particularly in low- and middle-income countries. In 2023, the World Health Organization (WHO) estimated 1.3 million new cases of TB in children aged under 15 years, accounting for approximately 12% of the global burden of TB (1). Although pulmonary TB is the most common presentation, 15%–20% of pediatric TB cases are extrapulmonary tuberculosis (EPTB) (2, 3). Among EPTB, abdominal tuberculosis (ATB) is a clinically significant yet not fully recognized form, accounting for approximately 6% of all TB cases. Among the various forms of EXPT, ATB is an important contributor, especially in high-burden regions, such as South Asia and sub-Saharan Africa (2). Despite these numbers, the true prevalence of pediatric ATB may be underestimated due to insufficient reporting, diagnostic challenges, and lack of standardized case definitions (4, 5).

ATB, a form of EXPT, is the sixth most common site of extrapulmonary involvement and may affect the gastrointestinal tract, peritoneum, mesenteric lymph nodes, and occasionally solid organs like the liver, spleen, and pancreas; ATB continues to pose significant diagnostic challenges due to its wide spectrum, including acute, subacute, and chronic manifestations and non-specific symptoms that often mimic other intra-abdominal pathologies (6–8). This challenge is not limited to the pediatric population. Studies in adults have similarly emphasized the frequent misdiagnosis of abdominal or gastrointestinal tuberculosis. The disease's ability to affect multiple organs and present with a diverse array of symptoms adds to the complexity of diagnosis (9).

In pediatric populations, ATB frequently presents with vague, non-specific symptoms, such as chronic abdominal pain, distension, fever, and weight loss, which are common to a wide range of abdominal conditions. Peritoneal and nodal forms predominate in children and are particularly challenging to diagnose (10). This overlap with more common surgical and inflammatory conditions, such as appendicitis, intestinal obstruction, peritonitis, and Crohn's disease, can lead to frequent misdiagnosis, delayed treatment, and unnecessary surgical interventions (11). Such diagnostic errors not only subject children to potentially avoidable procedures, such as appendectomy or laparotomy, but also delay the initiation of appropriate anti-tubercular therapy, increasing the risk of complications like intestinal perforation, abscesses, and chronic morbidity (12, 13).

The limited specificity of constitutional symptoms further complicates early identification. Low-grade fever, anemia, malaise, night sweats, and weight loss are present in only approximately one-third of pediatric ATB cases, making them unreliable as early diagnostic indicators (12). The diagnostic dilemma is particularly evident in cases where abdominal distension, ascites, or palpable masses are present features that, without a high index of suspicion, are easily mistaken for other surgical emergencies.

Misdiagnosis of abdominal tuberculosis in children is not only common but has serious clinical implications. Studies indicate that ATB is frequently mistaken for conditions such as inflammatory bowel disease (IBD), gastroenteritis, or surgical abdomen, resulting in delayed diagnosis and inappropriate management strategies (7, 12). Such delays are associated with higher morbidity and, in some cases, mortality due to disease progression and the development of complications (13, 14).

Given these challenges, this systematic review and meta-analysis aimed to examine the clinical characteristics contributing to the initial misdiagnosis or delay in diagnosis of ATB in children. By synthesizing evidence across the literature, we sought to identify key clinical features and risk factors that may mislead clinicians during the initial assessment. Our objective is to enhance clinical recognition of ATB in children, reduce diagnostic delays, and improve treatment outcomes through better-informed decision-making.

## Methods

This systematic review and meta-analysis were conducted and reported following the Preferred Reporting Items for Systematic Reviews and Meta-Analysis (PRISMA) 2020 guidelines (15). The study design, literature search, data extraction, and reporting processes adhered to these guidelines to ensure methodological rigor and transparency. A PRISMA flow diagram was used to illustrate the study selection process, including the number of records identified, and the screening, inclusion, and exclusion of studies.

### Literature search

A comprehensive systematic search was conducted across multiple databases, including PubMed, Web of Science, and Google Scholar, covering literature published between 1900 and 2024. The search strategy was developed using the Boolean operator and the following keywords and search terms: “children” OR “adolescent” AND “abdominal” OR “peritoneal” OR “intestinal” AND “tuberculosis” “case series,” and “case report.” To enhance coverage, the reference lists of selected articles were manually searched to identify additional relevant studies that might have been missed during the initial database search.

### Eligibility criteria

Studies were included if they met the following criteria: (1) case series involving pediatric patients (aged 0–18 years) diagnosed with ATB; (2) diagnosis confirmed through clinical, radiological, or microbiological evidence; (3) studies that specifically reported on misdiagnosis or delay in diagnosis, and described patients initially misdiagnosed with other conditions and later confirmed to have ATB; and (4) articles published in English.

Studies were excluded if they were case-control, cohort, randomized controlled trials (RCTs), or non-RCTs that did not present data relevant to misdiagnosis or delay in diagnosis. In addition, case reports without aggregated or comparative data, studies that did not report clinical features, and publications in languages other than English were excluded.

### Definition of key outcomes

Misdiagnosis was defined as a case where ATB was initially diagnosed as a different condition before the correct diagnosis was confirmed. Diagnostic delay was defined as a clinically relevant time gap between initial presentation and definitive diagnosis of ATB. However, due to the absence of consistent and quantifiable time frame data in the included studies, we were unable to differentiate between case and misdiagnosis and diagnostic delay. Cases with either misdiagnosis or diagnostic delay were categorized into the “misdiagnosis” group for pooled analysis.

Case series and case reports often contain incomplete data, particularly on clinical characteristics and diagnostic timelines. In this review, incomplete or unclear data were excluded from the meta-analysis. Clinical characteristics that could not be reliably classified as present or absent were omitted to avoid misclassification bias. Due to the retrospective and historical nature of most included studies, authors were not contacted for missing data. No imputation was performed, and only clearly reported, extracted data were included in the pooled analysis. We acknowledge that this conservative handling of missing data may have reduced the sample size for individual comparisons, potentially affecting the statistical power of some estimates.

### Screening and selection process

Screening and selection: title and abstract screening were carried out by two independent reviewers (MS and AK) to identify potentially relevant studies. Data analysis and interpretation were performed by MS, WX, and ZY. MJS and WX drafted the initial manuscript. LS and WC provided critical revisions and approved the final version. Data were extracted regarding the first author's name, year of publication, study design, number of participants in misdiagnosed and non-misdiagnosed groups, and patient demographics (age, gender).

### Quality assessment and outcome measurement

All included studies were retrospective in design and addressed research questions relevant to the objective of this review. Specifically, if a study reports of misdiagnosis or delay in diagnosis and provides detailed information on the clinical features that led to the initial misdiagnosis or diagnostic delay, it was included in the study. The extracted outcome analyzed was the clinical characteristics with misdiagnosis with the intent to identify patterns that may have led clinicians to incorrect or delayed diagnosis. Additional data, including the frequency of misdiagnosis and diagnostic methods, were also extracted where available to provide context. The methodological quality of the included studies was assessed using the Newcastle-Ottawa Scale (NOS) (16), with results summarized in Supplementary Table S1. Only studies meeting a minimum quality threshold based on the NOS criteria were included in the final analysis to ensure validity and reduce bias.

### Statistical analysis

The outcome measure in this study was the presence of specific clinical characteristics associated with misdiagnosis or delay in diagnosis of ATB in children. All outcomes were dichotomous data. Statistical analysis was performed using the Cochrane Collaboration's Review Manager software (RevMan 5.4). Odds ratios (ORs) with 95% confidence intervals (CIs) were calculated for each clinical characteristic to determine its association with misdiagnosis. Heterogeneity across the included studies was assessed using the *I*^2^ statistic to determine the percentage of total variation in effect estimates due to heterogeneity rather than random chance. Heterogeneity was categorized as follows: low (<40%), moderate (30%–60%), substantial (50%–90%), and high (>75%). An *I*^2^ value >50% was considered statistically significant. A random-effects model was applied to outcomes with substantial heterogeneity; otherwise, a fixed-effect model was used. Potential publication bias was evaluated using funnel plots, which plotted the effect size of individual studies against their standard error. Asymmetry in the funnel plot was interpreted as suggestive of publication bias or small-study effects.

## Results

**Figure 1:**
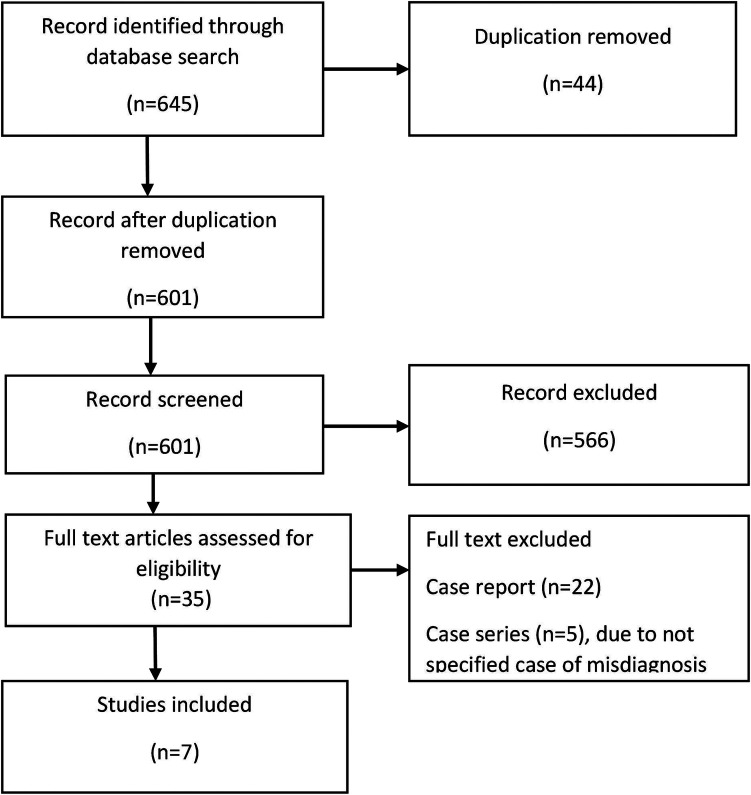
PRISMA flow diagram of study selection.

In this meta-analysis, a systematic search covering the period between 1900 and 2024 yielded a total of 645 studies from the databases (Figure 1). After removing 44 duplicate records, 601 studies were screened based on titles and abstracts. Of these, 566 records were excluded based on the predefined eligibility criteria (case reports without misdiagnosis details, reviews, editorials, letters, non-English publications, and unrelated topics).

Subsequently, 35 full-text articles were reviewed in detail. During this stage, 22 studies were excluded as they were individual case reports lacking analytical data, while five case series were excluded for not reporting outcomes related to misdiagnosis or delay in diagnosis. Ultimately, seven studies were included in the final meta-analysis.

### Characteristics of selected studies

The meta-analysis included only 60 cases of ATB from seven retrospective studies published between 1984 and 2023. Among these, 24 (40%) cases were identified as misdiagnosis or delay in diagnosis, and 36 (60%) cases were non-misdiagnosed. All included studies were case series or small observational studies. Given the small sample size, the statistical power to detect significant differences between groups was inherently low, increasing the risk of type II errors. The studies represented data from various countries, with clinical information retrieved and categorized based on the type of ATB (e.g., peritoneal or intestinal) and acuity of presentation. Study characteristics and key findings are summarized in Tables 1 and 2. These classifications were based on whether the diagnosis of ATB was missed or delayed at the time of initial presentation.

**Table 1:** Characteristics of the included studies.

Authors	Total patients number	Misdiagnosis	Not misdiagnosis	Male/female	Mean age	Study type	Country	Newcastle–Ottawa scale (NOS)
Lina et al., 2010 (17)	10	7	3	4/6	14.7	Case series	Taiwan	5
Delisle et al., 2015 (13)	6	3	3	0/6	12.5	Case series	Canada	5
Tinsa et al., 2010 (14)	13	4	9	3/10	9.8	Case series	Tunisia	5
Wong et al., 2019 (18)	6	3	3	3/3	11.3	Case series	Singapore	5
Chahed et al., 2010 (19)	11	3	8	3/7	5.6	Case series	Tunisia	5
Lancella et al., 2023 (11)	5	3	2	2/3	12	Case series	Italy	5
Dinler et al., 2008 (20)	9	1	8	5/4	14.2	Case series	Turkey	5

**Table 2:** Outcomes of the included studies.

References	TB Exposure, MD/NMD	Fever, MD/NMD	Cough, MD/NMD	Anorexia, MD/NMD	Ascites, MD/NMD	Abdominal mass, MD/NMD	Diarrhea/Constipation, MD/NMD	Vomiting, MD/NMD	Weight loss, MD/NMD	Abdominal Distention, MD/NMD	Abdominal Pain, MD/NMD	Misdiagnosis/Not misdiagnosis
Lina et al.	3/3	4/3	1/2	0/0	3/2	0/0	0/0	0/0	1/2	1/0	3/3	7/3
Delisle et al.	2/0	2/2	1/0	0/2	0/0	1/1	0/1	2/1	0/2	0/0	1/3	3/3
Tinsa et al.	1/0	0/4	0/1	2/5	4/4	1/1	0/0	0/0	0/0	4/4	2/6	4/9
Wong et al.	2/1	2/3	1/1	1/0	0/0	0/0	1/1	0/1	2/3	0/1	0/1	3/3
Chahed et al.	0/1	2/5	0/0	2/6	1/1	0/3	1/4	0/0	2/4	2/2	2/6	3/8
Lancella et al.	0/1	2/0	0/0	1/1	0/0	0/0	0/2	0/2	1/1	0/0	3/1	3/2
Dinler et al.	1/6	0/4	½	0/0	0/0	0/0	0/0	0/0	0/3	1/8	1/5	1/8

Note: Dichotomous data (TB exposure, fever, cough, anorexia, ascites, abdominal mass, diarrhea/constipation, vomiting, weight loss, abdominal distension, and abdominal pain) are reported as the total number of patients with specific outcomes out of total patients.

MD, misdiagnosis; NMD, not misdiagnosis.

This meta-analysis aimed to assess whether specific clinical characteristics were associated with the misdiagnosis of ATB in children. Across the seven included studies, no individual symptoms demonstrated a statistically significant association with the likelihood of misdiagnosis. The pooled OR for each clinical variable reflected the absence of clear predictive value.

**Figure 2:**
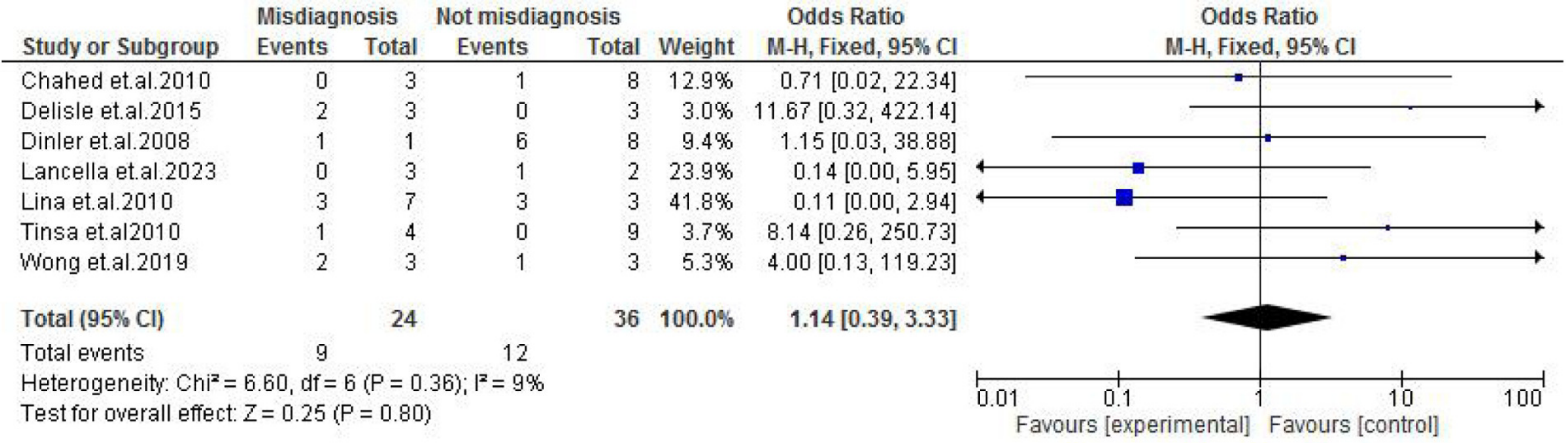
Forest plot of TB exposure comparing abdominal tuberculosis (ATB) misdiagnosis vs. not misdiagnosis.

**Figure 3:**
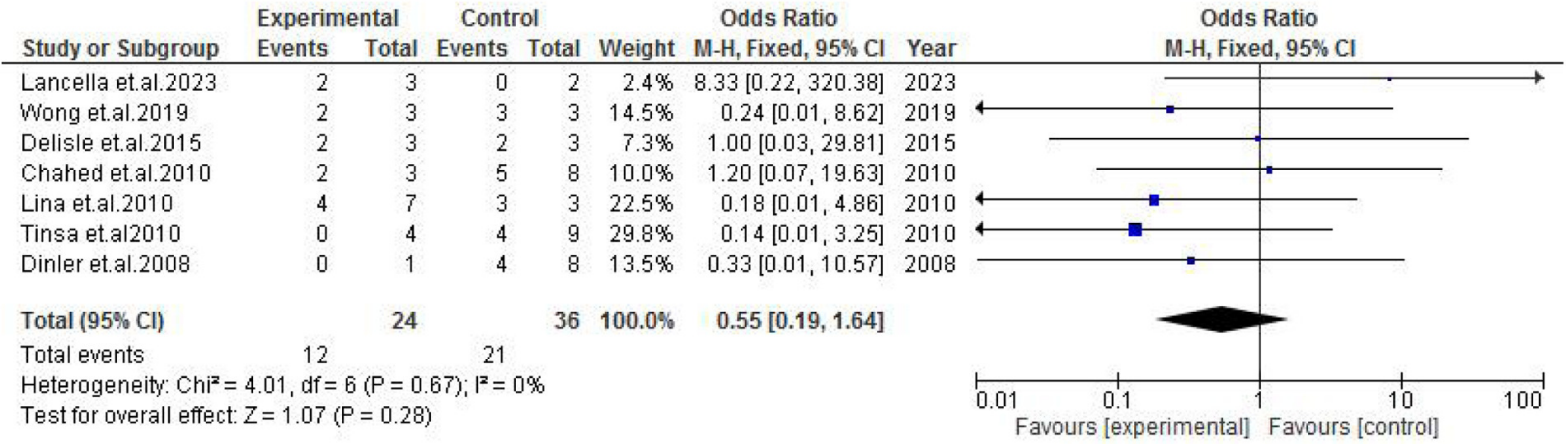
Forest plot of fever comparing abdominal tuberculosis (ATB) misdiagnosis vs. not misdiagnosis.

**Figure 4:**
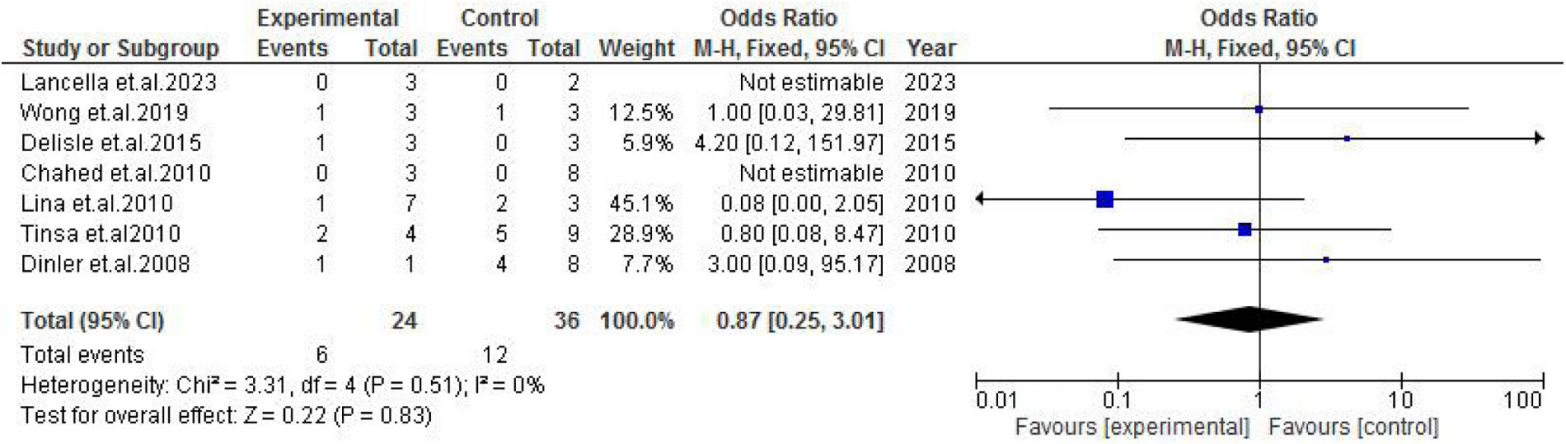
Forest plot of cough comparing abdominal tuberculosis (ATB) misdiagnosis vs. not misdiagnosis.

**Figure 5:**
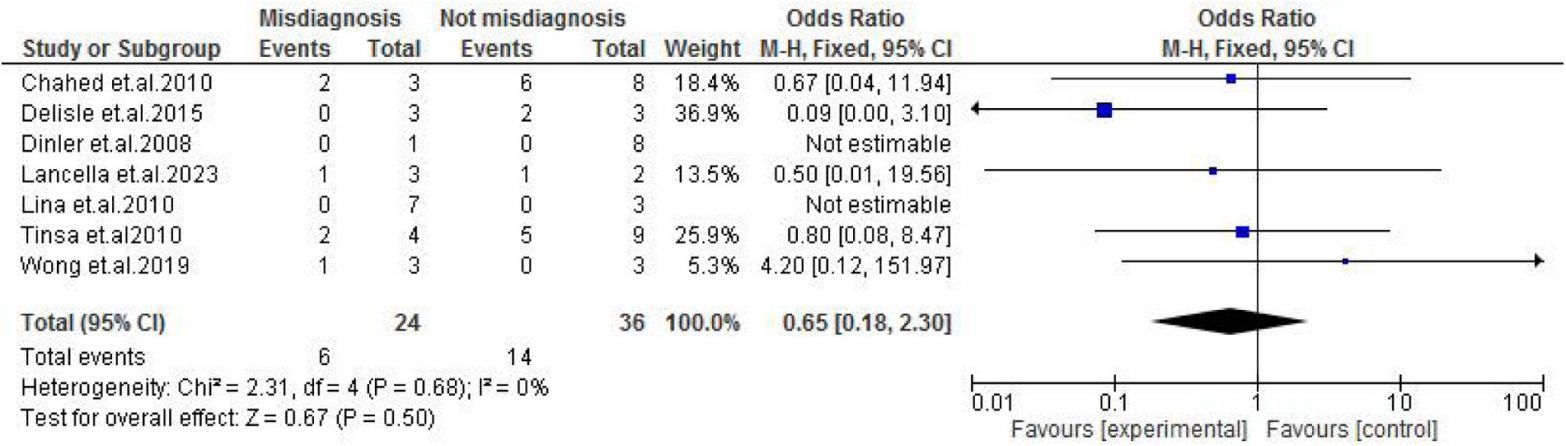
Forest plot of anorexia comparing abdominal tuberculosis (ATB) misdiagnosis vs. not misdiagnosis.

**Figure 6:**
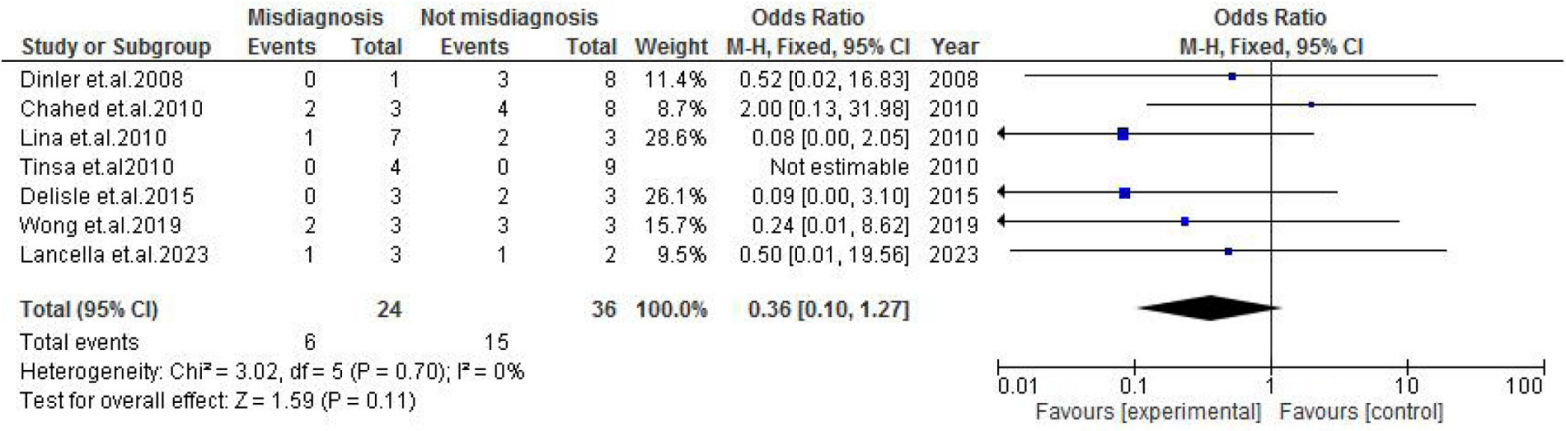
Forest plot of weight loss comparing abdominal tuberculosis (ATB) misdiagnosis vs. not misdiagnosis.

**Figure 7:**
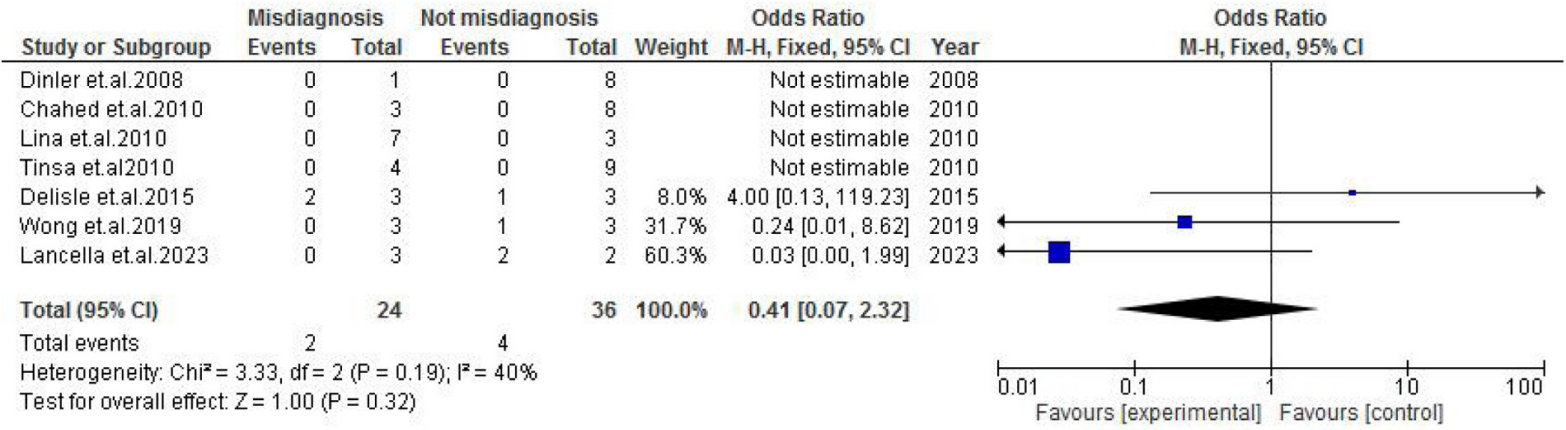
Forest plot of vomiting comparing abdominal tuberculosis (ATB) misdiagnosis vs. not misdiagnosis.

**Figure 8:**
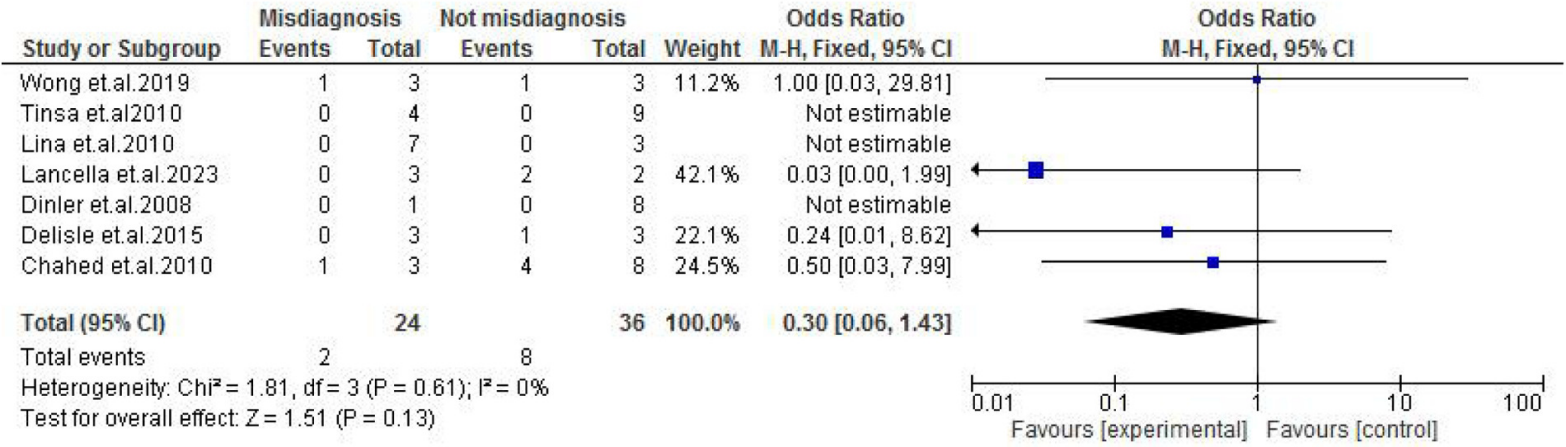
Forest plot of diarrhea/constipation comparing abdominal tuberculosis (ATB) misdiagnosis vs. not misdiagnosis.

TB exposure was not found to significantly increase the odds of misdiagnosis (OR: 1.14; 95% CI: 0.39–3.33; *p* = 0.80) (Figure 2), fever (OR: 0.55; 95% CI: 0.19–1.64; *p* = 0.28) (Figure 3), or cough (OR: 0.87; 95% CI: 0.25–3.01; *p* = 0.83) (Figure 4). Other constitutional and abdominal symptoms similarly showed no statistically significant associations, including anorexia (OR: 0.65; 95% CI: 0.18–2.30; *p* = 0.50) (Figure 5), weight loss (OR: 0.36; 95% CI: 0.10–1.27; *p* = 0.11) (Figure 6), vomiting (OR: 0.41; 95% CI: 0.07–2.32; *p* = 0.32) (Figure 7), and diarrhea or constipation (OR: 0.30; 95% CI: 0.06–1.43; *p* = 0.13) (Figure 8).

**Figure 9:**
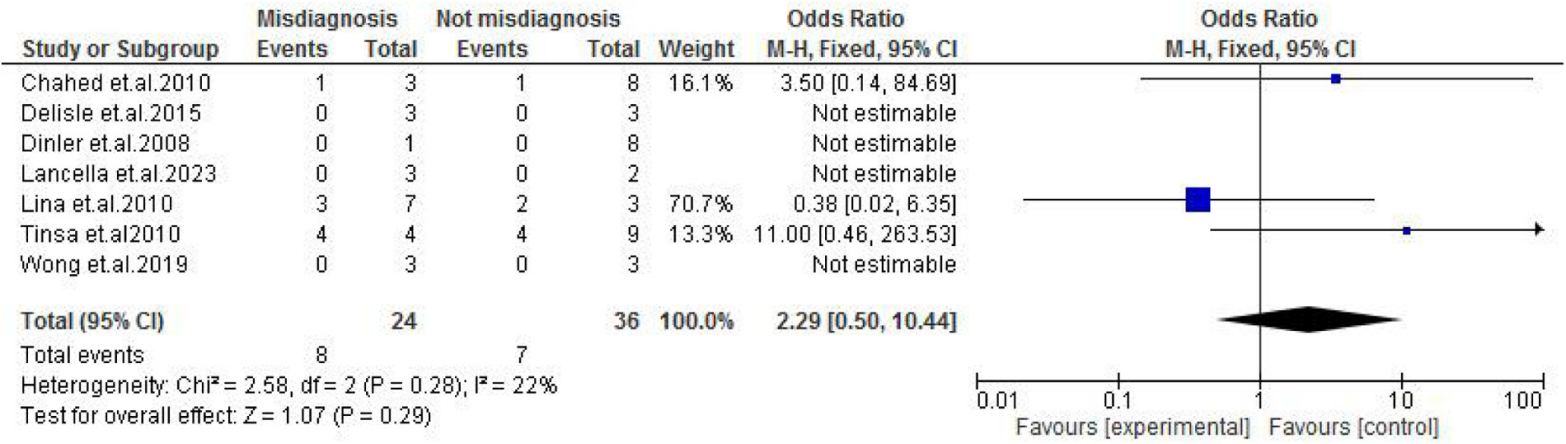
Forest plot of ascites comparing abdominal tuberculosis (ATB) misdiagnosis vs. not misdiagnosis.

**Figure 10:**
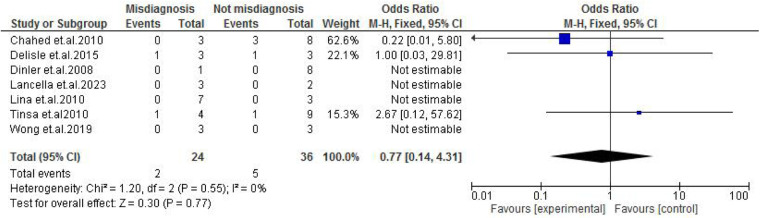
Forest plot of abdominal mass comparing abdominal tuberculosis (ATB) misdiagnosis vs. not misdiagnosis.

**Figure 11:**
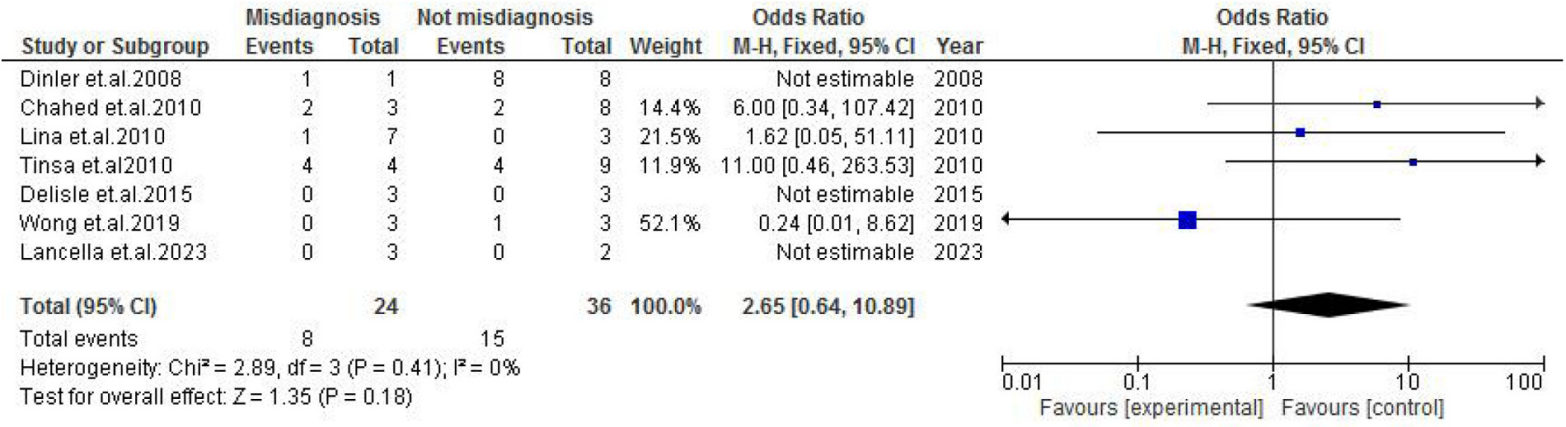
Forest plot of abdominal distension comparing abdominal tuberculosis (ATB) misdiagnosis vs. not misdiagnosis.

**Figure 12:**
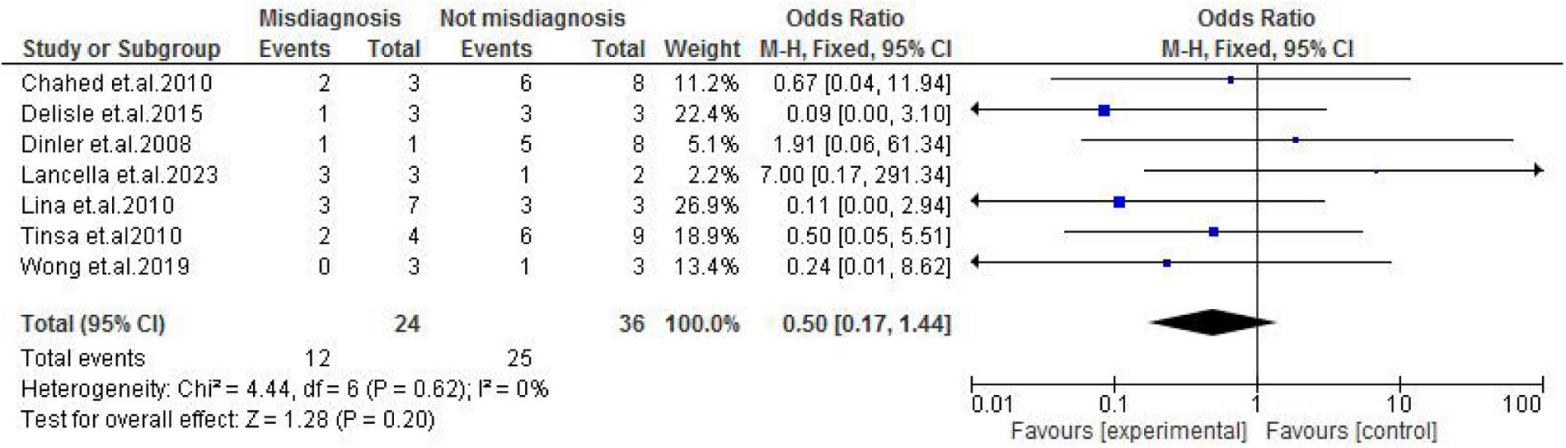
Forest plot of abdominal pain comparing abdominal tuberculosis (ATB) misdiagnosis vs. not misdiagnosis.

Abdominal findings, such as the presence of ascites (OR: 2.29; 95% CI: 0.50–10.44; *p* = 0.29) (Figure 9), abdominal mass (OR: 0.77; 95% CI: 0.14–4.31; *p* = 0.77) (Figure 10), abdominal distension (OR: 2.65; 95% CI: 0.64–10.89; *p* = 0.18) (Figure 11), and abdominal pain (OR: 0.50; 95% CI: 0.17–1.44; *p* = 0.20) (Figure 12), also did not reach statistical significance.

### Publication bias

Funnel plot analysis for each clinical characteristic is presented in the Supplementary File. Several plots demonstrated asymmetry, suggesting the presence of publication bias or heterogeneity potentially due to selective reporting or underrepresentation of smaller studies with non-significant results. Although most outcomes showed low heterogeneity, vomiting displayed moderate heterogeneity (*I*^2^ = 40%), and a random-effects model was applied. The overall low heterogeneity supports the consistency of the findings across studies.

## Discussion

The diagnosis of ATB in children remains particularly challenging due to its non-specific clinical presentation and overlap with other common pediatric abdominal conditions, which frequently leads to misdiagnosis or delayed diagnosis. However, ATB constitutes approximately 1%–3% of total TB cases and 12% of extrapulmonary TB cases in children under 15 years (21, 22).

ATB has been reported more frequently among children aged under 5 years, particularly in high-burden countries. However, recent studies from India, Taiwan, Turkey, and Tunisia suggest a shift toward older children and adolescents, with mean ages reported between 9.5 and 14.7 years (23, 24), suggesting evolving epidemiological patterns that may impact clinical suspicion thresholds. The wide clinical spectrum from chronic symptoms to acute surgical abdomen further complicates early recognition. In particular, the presence of comorbidities such as HIV or malnutrition may obscure classic features, delaying diagnosis and increasing the risk of complications (14).

This meta-analysis evaluated clinical characteristics observed in misdiagnosed cases of ATB in children. Ascites were more prevalent in the misdiagnosed group with an OR of 2.29 (*p* = 29), though a result was not statistically significant toward ascites being a misleading clinical characteristic. As a common feature in several gastrointestinal disorders, such as nephrotic syndrome or liver disease, ascites may divert clinical focus away from TB, especially in low-resource settings (14, 18). Similarly, abdominal distension was more frequent in misdiagnosed cases, with an OR of 2.65 (*p* = 0.18), reflecting its commonality in many gastrointestinal pathologies, such as obstruction, ileus, or severe malnutrition (17, 25). Although not statistically significant, this finding is consistent with the notion that such symptoms can mask ATB, especially when constitutional signs are mild or absent.

Interestingly, weight loss, often considered a hallmark of TB, was not protective against misdiagnosis in this cohort (OR: 0.50; *p* = 0.11). Despite its frequent occurrence in correctly diagnosed cases, its lack of specificity—given its presence in malnutrition, chronic infections, and malignancies—limits its diagnostic utility in distinguishing ATB from other causes of pediatric abdominal disease (26).

Overall, the analysis underscores that no single symptom reliably differentiates misdiagnosed from correctly diagnosed ATB cases. Common features such as abdominal pain, vomiting, fever, and anorexia were prevalent across both groups, emphasizing the necessity for a high index of suspicion, particularly in TB-endemic regions. Although trends suggested that ascites and abdominal distension may be more prevalent in misdiagnosed cases, the findings did not reach statistical significance, partly due to small sample sizes and heterogeneity among studies.

## Limitations

This study has several limitations. First, the incomplete and inconsistent reporting across primary studies limited the inclusion of comprehensive datasets, affecting statistical power. Second, we were unable to analyze the contribution of radiological or laboratory parameters, which are essential in diagnosing ATB. Third, the inclusion was restricted to case series, as other study designs did not report misdiagnosis-specific data. These limitations call for more robust, prospective research with standardized reporting and the inclusion of diverse study types to better understand and address diagnostic delays in pediatric ATB.

## Conclusion

This meta-analysis reinforces the complexity of diagnosing pediatric abdominal tuberculosis based on clinical presentation alone. Despite observing trends toward misdiagnosis with symptoms like ascites and abdominal distension, the lack of statistical significance limits their predictive value. The results highlight the critical need for clinical vigilance, early use of diagnostic investigations, and consideration of ATB in differential diagnoses for children presenting with unexplained abdominal symptoms in high-burden areas. Future research should aim to validate these findings in larger cohorts and integrate laboratory and imaging data to support earlier, more accurate diagnoses.

## Data Availability

The original contributions presented in the study are included in the article/Supplementary Material, further inquiries can be directed to the corresponding author.

## References

[1] WHO (2023). Tuberculosis. Available online at: https://www.who.int/news-room/fact-sheets/detail/tuberculosis (Accessed November 07, 2023).

[2] Jain SK Ordonez A Kinikar A Gupte N Thakar M Mave V Pediatric tuberculosis in young children in India: a prospective study. Biomed Res Int. (2013) 2013:783698. 10.1155/2013/783698 24386640 PMC3872373

[3] Barberis I Bragazzi NL Galluzzo L Martini M. The history of tuberculosis: from the first historical records to the isolation of Koch’s bacillus. J Prev Med Hyg. (2017) 58(1):E9–12. 28515626 PMC5432783

[4] Huang G Wu KK Li XN Kuai JH Zhang AJ. Intestinal tuberculosis with small bowel stricture and hemorrhage as the predominant manifestation: three case reports. World J Gastrointest Surg. (2024) 16(1):248–56. 10.4240/wjgs.v16.i1.248 38328313 PMC10845280

[5] Çay Ü Gündeşlioğlu ÖÖ Alabaz D. Evaluation of cases with abdominal tuberculosis in children: ten years of experience from a single center in Turkey. South Clin Ist Euras. (2022) 33(3):298–303.

[6] Sharma M Bhatia V. Abdominal tuberculosis. Indian J Med Res. (2004) 120:305–15. 15520484

[7] Desai CS Josh AG Abraham P Desai DC Deshpande RB Bhaduri A Hepatic tuberculosis in absence of disseminated abdominal tuberculosis. Ann Hepatol. (2006) 5(1):41–3. 10.1016/S1665-2681(19)32038-1 16531964

[8] Kilborn T Wieselthaler N Nondela B Cox S. Abdominal tuberculosis in children—imaging and intervention. J Pediatr Infect Dis. (2017) 12(1):89–98. 10.1055/s-0037-1599122

[9] Kudu E Danış F. Recognizing and addressing the challenges of gastrointestinal tuberculosis. World J Clin Cases. (2024) 12(19):3648–53. 10.12998/wjcc.v12.i19.3648 38994296 PMC11235435

[10] Cruz AT Starke JR. Clinical manifestations of tuberculosis in children. Paediatr Respir Rev. (2007) 8(2):107–17. 10.1016/j.prrv.2007.04.008 17574154

[11] Lancella L Abate L Cursi L Chiopris G Nicoletti L Principi N Abdominal tuberculosis in children: a case series of five patients. Microorganisms. (2023) 11(3):730. 10.3390/microorganisms11030730 36985303 PMC10054026

[12] Usta M Urganci N Dalgic N Kızılkan NU Kurtaraner T Karadag CA. Clinical presentation in a series of eight children with abdominal tuberculosis: experience of a single-center in Turkey. Iran J Pediatr. (2017) 27(6). 10.5812/ijp.9766

[13] Delisle M Paediatric abdominal tuberculosis in developed countries: case series and literature review. Arch Dis Child. (2016) 101(3):253–8. 10.1136/archdischild-2015-308720 26699532

[14] Tinsa F Essaddam L Fitouri Z Brini I Douira W Ben Becher S Abdominal tuberculosis in children. J Pediatr Gastroenterol Nutr. (2010) 50(6):634–8. 10.1097/MPG.0b013e3181b6a57b 20386326

[15] Page MJ McKenzie JE Bossuyt PM Boutron I Hoffmann TC Mulrow CD The PRISMA 2020 statement: an updated guideline for reporting systematic reviews. BMJ. (2021) 372:n71. 10.1136/bmj.n71 33782057 PMC8005924

[16] Stang A. Critical evaluation of the Newcastle-Ottawa scale for the assessment of the quality of nonrandomized studies in meta-analyses. Eur J Epidemiol. (2010) 25(9):603–5. 10.1007/s10654-010-9491-z 20652370

[17] Lin Y-S Huang Y-C Lin T-Y. Abdominal tuberculosis in children: a diagnostic challenge. J Microbiol Immunol Infect. (2010) 43(3):188–93. 10.1016/S1684-1182(10)60030-8 21291845

[18] Wong SA Lee Meijuan D Loh SW Thoon KC Tan NWH Chong CY. Pediatric abdominal tuberculosis in Singapore: a 10-year retrospective series. Glob Pediatr Health. (2020) 7:2333794X20903952. 10.1177/2333794X20903952 32076630 PMC7003167

[19] Chahed J Mekki M Mansour A Ben Brahim M Maazoun K Hidouri S Contribution of laparoscopy in the abdominal tuberculosis diagnosis: retrospective study of about 11 cases. Pediatr Surg Int. (2010) 26(4):413–8. 10.1007/s00383-010-2555-z 20162421

[20] Dinler G Sensoy G Helek D Kalayci AG. Tuberculous peritonitis in children: report of nine patients and review of the literature. World J Gastroenterol. (2008) 14(47):7235–9. 10.3748/wjg.14.7235 19084940 PMC2776883

[21] Shah I Uppuluri R. Clinical profile of abdominal tuberculosis in children. Indian J Med Sci. (2010) 64(5):204–9. 10.4103/0019-5359.98935 22842319

[22] Tobin EH Khatri AM. Abdominal Tuberculosis. In: StatPearls. Treasure Island (FL): StatPearls Publishing (2025). 32310575

[23] Kilic O Somer A Hançerli Törün S Keser Emiroğlu M Salman N Salman T Assessment of 35 children with abdominal tuberculosis. Turk J Gastroenterol. (2015) 26(2):128–32. 10.5152/tjg.2015.6123 25835110

[24] Saczek KB Schaaf HS Voss M Cotton MF Moore SW. Diagnostic dilemmas in abdominal tuberculosis in children. Pediatr Surg Int. (2001) 17(2–3):111–5. 10.1007/s003830000455 11315266

[25] Lal SB Bolia R Menon JV Venkatesh V Bhatia A Vaiphei K Abdominal tuberculosis in children: a real-world experience of 218 cases from an endemic region. JGH Open. (2020) 4(2):215–20. 10.1002/jgh3.12245 32280767 PMC7144780

[26] Sanai FM Bzeizi KI. Systematic review: tuberculous peritonitis–presenting features, diagnostic strategies and treatment. Aliment Pharmacol Ther. (2005) 22(8):685–700. 10.1111/j.1365-2036.2005.02645.x 16197489

