# Supplementary material for: Clinical characteristics leading to misdiagnosis of abdominal tuberculosis in children: a systematic review and meta-analysis
Source: Front Pediatr. 2025 Sep 2;13:1616608. doi: 10.3389/fped.2025.1616608 (PMC12439471; doi:10.3389/fped.2025.1616608)
Supplement: Supplementary file 1 [file Datasheet1.docx]

Supplementary Material

**Table 1: Newcastle– Ottawa Quality Assessment Scale for Cohort Studies**

| Authors | Represent-ativeness of the exposed cohort (1point) | Selection of the non-exposed cohort | Ascertain-ment of exposure | Demonst-ration that outcome not present at start | Compara-bility of cohort  (0 points) | | Assessment of Outcome | Follow-up length adequate for outcome to occur | Adequacy of Follow-up of cohorts (accounted for non-index hospitals) | Total Score  (9 points possible) | |
| --- | --- | --- | --- | --- | --- | --- | --- | --- | --- | --- | --- |
| Lina et al., 2010 | 1 | 0 | 1 | 1 | 0 | 1 | | 1 | 1 | | 5 |
| Delisle et al., 2015 | 1 | 0 | 1 | 1 | 0 | 1 | | 1 | 1 | | 5 |
| Tinsa et al., 2010 | 1 | 0 | 1 | 1 | 0 | 1 | | 1 | 1 | | 5 |
| Wong et al., 2019 | 1 | 0 | 1 | 1 | 0 | 1 | | 1 | 1 | | 5 |
| Chahed et al., 2010 | 1 | 0 | 1 | 1 | 0 | 1 | | 1 | 1 | | 5 |
| Lancella et al., 2023 | 1 | 0 | 1 | 1 | 0 | 1 | | 1 | 1 | | 5 |
| Dinler et al., 2008 | 1 | 0 | 1 | 1 | 0 | 1 | | 1 | 1 | | 5 |


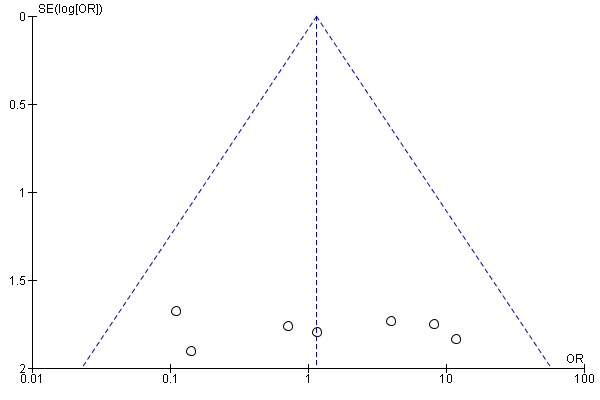

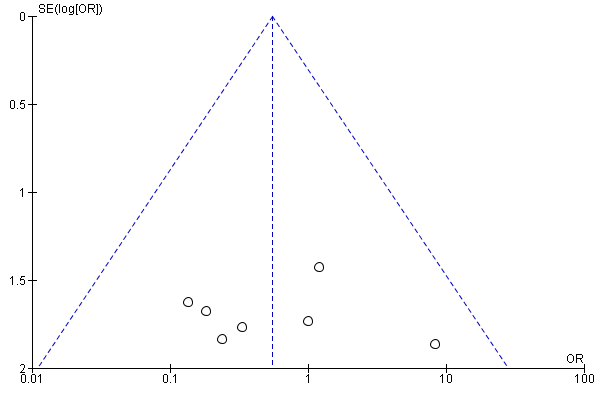

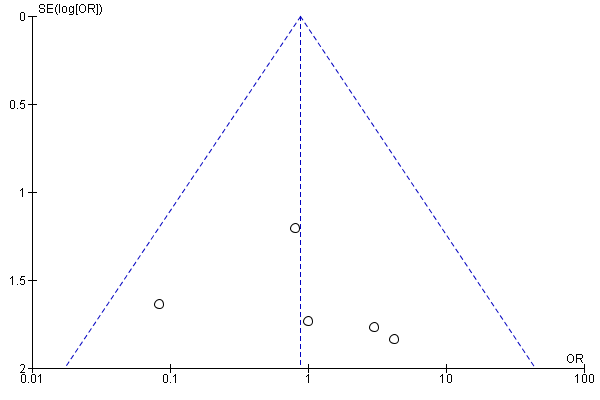


F

E

C

B

A


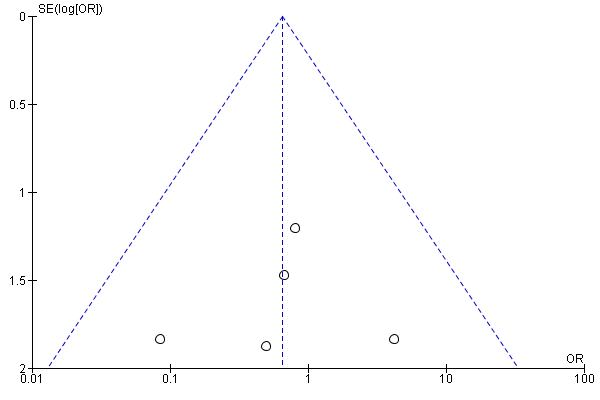

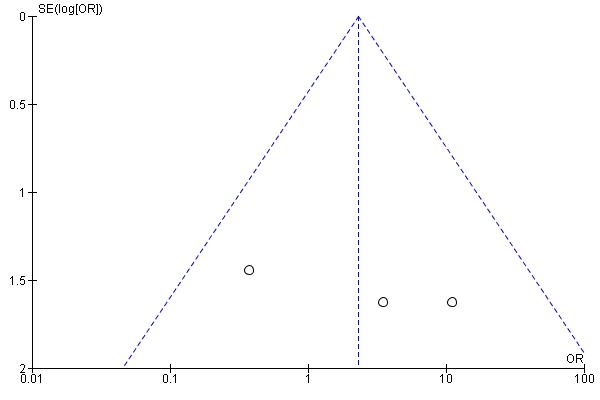

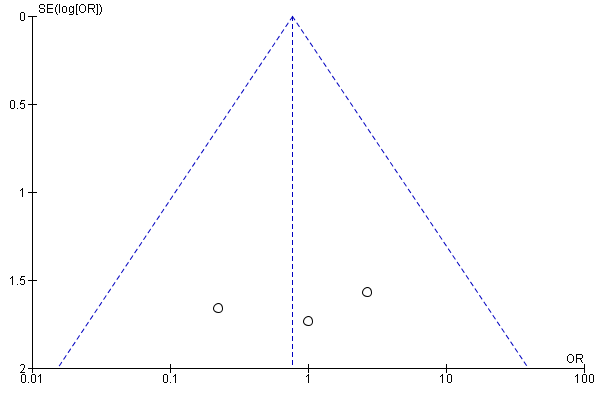


D

**
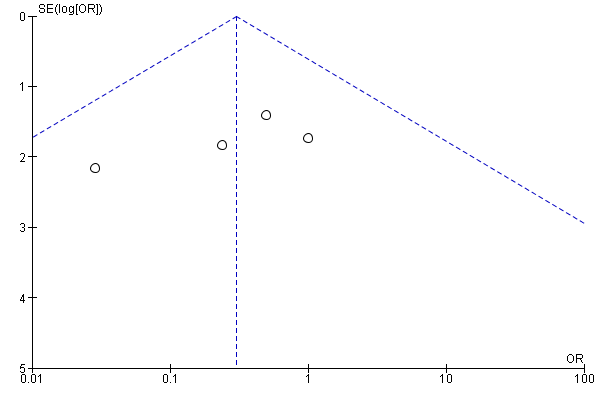

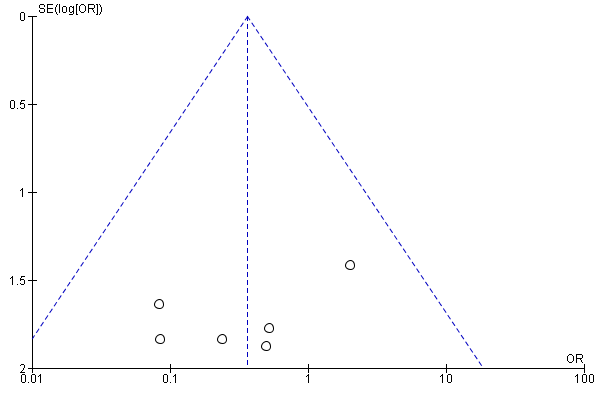

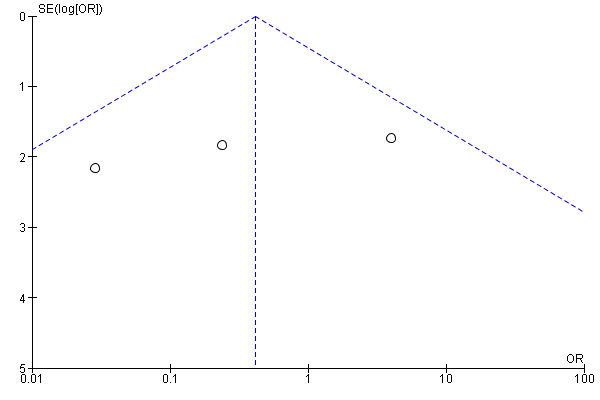
**

I

H

G

**
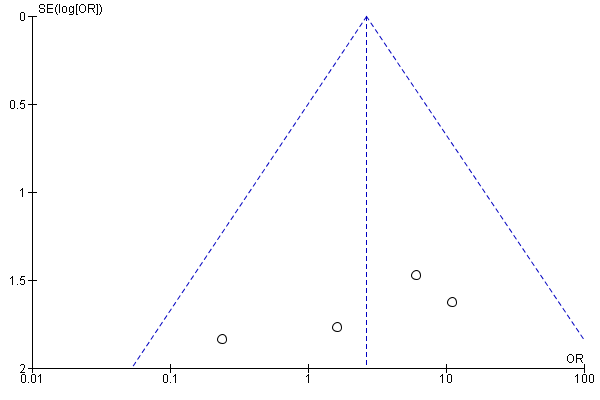

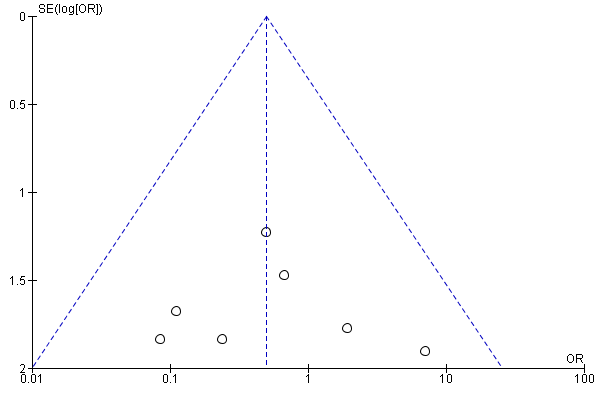
**

K

J

**Figure 13**: **Funnel plot analysis**: **A**. TB exposure, **B.** Fever, **C**. Cough, **D**. Anorexia, **E**. Ascites, **F**. Abdominal mass, **G**. Diarrhea/Constipation, **H**. Vomiting, **I**. Weight loss, **J**. Abdominal distension and **K**. Abdominal p
